# Effectiveness of cannabis use and cannabis use disorder interventions: a European and international data synthesis

**DOI:** 10.1007/s00406-024-01829-5

**Published:** 2024-05-23

**Authors:** Jason P. Connor, Jakob Manthey, Wayne Hall, Daniel Stjepanović

**Affiliations:** 1 National Centre for Youth Substance Use Research, The University of Queensland, St Lucia, Australia; 2 Discipline of Psychiatry, School of Medicine, The University of Queensland, Herston, Australia; 3 Center for Interdisciplinary Addiction Research (ZIS), Department of Psychiatry and Psychotherapy, University Medical Center Hamburg-Eppendorf (UKE), Martinistraße 52, 20246 Hamburg, Germany; 4 Department of Psychiatry, Medical Faculty, University of Leipzig, Semmelweisstraße 10, 04103 Leipzig, Germany; 5 Queensland Alliance for Environmental Health Sciences, The University of Queensland, Woolloongabba, Australia; 6 School of Psychology, The University of Queensland, St Lucia, Australia

**Keywords:** Cannabis, Cannabis use disorder, Treatment, Psychotherapy

## Abstract

This data synthesis examined the effectiveness of behavioural and pharmacological approaches for cannabis treatment. We integrated findings from high level evidence studies and prioritised data from Europe when available. The synthesis found that only a relatively small number of published behavioural and pharmacological studies on cannabis interventions have been conducted in Europe. Applying both European and non-European data, it was found that Cognitive Behavioural Therapy (CBT) and/or Motivational Enhancement Therapy (MET) improved short-term outcomes in the frequency of cannabis use and dependency severity, although abstinence outcomes were less consistent. These improvements were typically not maintained nine months after treatment. CBT and MET (or combined CBT + MET) treatments that extend beyond four sessions were more effective than fewer sessions over a shorter duration. Combining CBT or MET (or combined CBT + MET) with adjunctive Contingency Management (CM) improved therapeutic outcomes. No pharmacotherapies have been approved for the management of cannabis use, cannabis use disorders or cannabis withdrawal. Despite only weak evidence to support the use of pharmacological agents, some are used ‘off-label’ to manage withdrawal symptoms outside clinical trials.

## Introduction

It is estimated 27.4% of adults (aged 15–64) in the European Union have used cannabis in their lifetime and 15.4% (ranging from 3.4 to 21.8% in member states) of 15–34 year-olds have used cannabis in the past year, based on most recent survey data [1]. Comparatively, approximately 3.9% of the global adult population has used cannabis in the past 12 months [2], with rates highest in Western Europe, North America, Oceania, West and Central Africa. In developed countries, most cannabis users initiate cannabis use in late adolescence, with the median onset age in the Americas, Europe, Asia, New Zealand, the Middle East and Africa at 18–19 years (mean 15–16 years) [3, 4]. Approximately 1 in 10 cannabis users develop cannabis use disorder (CUD) [2, 5]. In Europe, it is estimated that around 1.8% of adults in the European Union are daily or almost daily cannabis users [1]. Meta-analyses of the existing literature find that daily use and younger initiation of cannabis use greatly increase the risks of developing CUD [6, 7]. The peak age of CUD onset is 19.5 years [8]. Over half (61%) of daily users are under the age of 35 and around three-quarters are male [1].

Many young adults cease cannabis use and mature out of CUD without formal treatment as they enter the labour market, find a partner, and take responsibility for child rearing [9–12]. In a large longitudinal German study of young, regular cannabis users (14–24 years), 44% had ceased cannabis use by the 4 year follow-up and 54% were not using after 10 years [13]. Effective treatments are available for those whose CUDs do not remit without treatment, doubling abstinence rates in the short-term compared with non-active treatment [14]. This is particularly important in Europe where cannabis is the most widely used illicit drug, peak past year use occurs in the 15–24 age group (19.2%), and cannabis use disorders account for 35% of all treatment demand for problems linked to illicit drug use [1].

## Therapies for cannabis use and cannabis use disorders

### Behavioural therapies

**Fig. 1:**
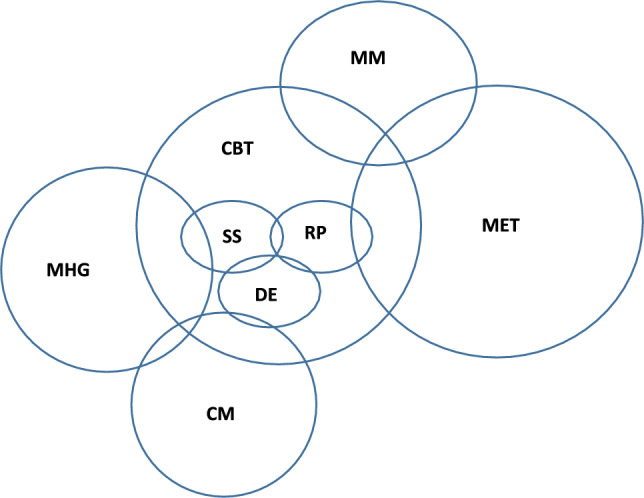
Conceptual overlap between behavioural interventions for cannabis use and CUD. Cognitive-Behavioural Therapy (CBT), Motivational Enhancement Therapy (MET) including brief MET (bMET), Combined CBT + MET (CBT + MET), Mindfulness Meditation (MM), Contingency Management (CM), Social Support counselling (SS), Drug Education counselling (DE), Relapse Prevention (RP), Mutual Help Groups (MHG), based on the 12-step approaches

There is considerable conceptual and theoretical overlap between different psychosocial interventions for CUD (Fig. 1). The most widely researched behavioural treatments for problem cannabis use and CUD are Cognitive-Behavioural Therapy (CBT) and Motivational Enhancement Therapy [MET; 15, 16].

CBT targets dysfunctional thoughts (cognition) and actions (behaviour) that have been identified as triggers for cannabis use and maintenance of use. Key CBT strategies include enhancing problem-solving skills, developing more effective coping strategies, and relaxation approaches. Motivational Enhancement Therapy (MET), including brief MET, promotes an empathic, respectful, and non-judgmental therapeutic relationship between therapist and cannabis user. It assists patients to resolve ambivalence and set goals to modify cannabis use. MET can be offered in a briefer form (bMET) that is typically only one or two sessions in duration. In practice, CBT and MET are often combined, with MET typically used in the earlier stages of treatment to enhance treatment engagement [15, 16].

Social Support (SS) skills, Drug Education (DE) and Relapse Prevention (RP), although offered as stand-alone treatments, can also be included as components of CBT and other behavioural treatments. SS includes pragmatic strategies that aim to enhance social support across work, educational and personal domains to support cannabis use goals. DE incorporates evidence-based information on the health risks associated with cannabis use to challenge dysfunctional or inaccurate thoughts and behaviours, and may include strategies to reduce use. RP assists the person to understand that CUD is a chronic, relapsing condition and helps them to adopt strategies that reduce relapse. High risk situations for cannabis use are identified for each patient and effective problem-solving, relaxation and assertion skills employed to minimise risk of relapse to previous levels of cannabis use [15]. RP can be applied independently but is also a key feature of MET, where relapse is considered an important stage in the change process and is used as an opportunity to learn about more effective ways to sustain the original treatment goals [17, 18].

Mindfulness Meditation (MM), often described as a ‘mind-and-body’ approach, typically examines ‘here-and-now’ experiences and images to identify and manage negative cognitions which can be patient-directed or guided by a therapist. MM also targets negative symptoms of cannabis use and withdrawal, such as irritability, anger, depression, anxiety; and which may be improved through meditative practices [19]. Rather than challenging irrational or dysregulated beliefs as occurs in CBT, MM encourages patients to release negative thoughts without challenging, achieving similar aims as CBT to reduce the preoccupation with substance use and substance craving. MM and MET have some complementary mechanisms, for example by increasing interoceptive awareness of substance use on the patient’s physical and emotional health.

Contingency Management (CM) uses money or vouchers as incentives (reinforcers) to increase treatment compliance and cannabis use goals. CM strategies utilise positive reinforcement that rewards positive change over approaches that punish or remove incentives for failure to meet treatment goals. An important component of CM is drug education (DE) relating to the risk of ongoing substance use and treatment compliance. CM to date has been largely used in clinical research trials as an adjunct to CBT, MET or CBT + MET [15, 16].

Mutual Help Groups (MHGs) are typically based on 12-step approaches. The most widely recognised cannabis-focused mutual peer support group is Marijuana Anonymous (MA). MA groups have an abstinence goal and typically work through the 12 steps of recovery used by Alcoholics Anonymous. A sponsor is typically allocated to new members to support abstinence outside meetings. Trained therapists are rarely engaged in community-based MHGs, with sponsors who have sustained a period of abstinence, combined with peers with common cannabis use goals, typically facilitating meetings. A key function of these groups is social support, and therefore their content overlaps with the conceptually narrower SS interventions [15, 20].

Family Therapy (FT) leverages therapeutic approaches that span across many of the aforementioned theoretical approaches [21]. FT is defined primarily by the participants in therapy as opposed to the theoretical framework, typically incorporating a family unit rather than focusing on individuals. Therefore, for the purposes of this synthesis of RCTs, FT does not appear in Fig. 1 but studies using FT have been summarised in Table 1.

**Table 1:** Characteristics and Key Findings of Behavioural Interventions for Cannabis Use and CUD drawn from Systematic Reviews and Meta-analyses

Therapy	Study and participant details	Therapy sessions and completion	Cannabis use demographics	Strength of evidence
Cognitive-Behavioural Therapy **(CBT)** including brief CBT **(bCBT)** and group CBT **(gCBT)**	Five trials utilised CBT, 2 trials examined the effectiveness of gCBT, and 1 implemented bCBT No trials were conducted in Europe. Six trials (4 CBT and 2 gCBT) were conducted in the USA, and 2 in Australia (1 CBT, 1 bCBT) Most trials (50.00%; 2 CBT; 1 bCBT; 1 gCBT) restricted recruitment to adults. The remaining trials recruited either only adolescents (37.50%; 2 CBT, 1 gCBT) or both adolescents and adults (12.50%, 1 CBT) The participants across trials were predominantly male (77.32%), and an average 25.67 years old (average 20.70 years for CBT, 32.30 for brief CBT, and 29.63 for gCBT)	One CBT trial did not report number of sessions planned or delivered. The remaining trials planned 10–14 sessions (*M* and median = 12.00). On average, 66.83% of sessions were delivered (*M* = 7.95 and median = 8.20 sessions) Trials utilising gCBT planned 14–32 sessions (*M* = 14.00 and median = 23), with participants attending an average of 66.07% of planned sessions A single trial examined bCBT, contrasting a single session against six. Those assigned the longer six session treatment were less likely to attend (70.00% of sessions) compared to those assigned the shorter single session (87.80% completed) Duration ranged from 50–120 min per session across the trials. CBT sessions were typically one hour in duration (*M* = 62 median = 60 min) and were shorter in duration than gCBT (*M* and median = 105 min)	Half of trials included cannabis use or disorder as an inclusion criterion. Those that did not either recruited individuals with substance use more widely, or focussed on cannabis users but did not report a cannabis use or diagnosis inclusion criterion Across studies, the majority (91.96%) of the sample met criteria for a diagnosis relating to cannabis use Trial participants reported using cannabis on an average of 19.43 (median = 20.55; range 9.1–26.08) of the past 28 days A minority of trials (42.86%) reported the age of first cannabis use, which was an average of 15.22 years (range 14.73–15.93)	***Strong Evidence:*** CBT is effective in reducing frequency of cannabis use and dependence severity, although fewer studies have examined abstinence as a primary treatment outcome and results are less consistent [14, 26, 30]. When follow-up was extended, CBT treatment benefits were not maintained at six to nine months post intervention [14] As only a single trial [33] examined the effectiveness of briefer forms of CBT, it is premature to draw conclusions of the effectiveness of bCBT
Motivational Enhancement Therapy **(MET)** including brief MET **(bMET)**	More trials (k = 17) utilised bMET rather than lengthier MET (k = 7) interventions The majority of trials (3 MET and 12 bMET) were conducted in the USA. Five trials were conducted in Europe (2 MET and 3 bMET), and one trial in each of Canada (MET), Brazil (bMET) and Chile (bMET) Just over half of all trials restricted their sample to adults (52.17%; 5 MET and 7 bMET). Only 8.70% of all trials (both bMET) recruited an exclusively adolescent sample. The remaining trials (39.13%; 1 MET and 8 bMET) recruited both adolescents and adults The majority of participants across MET and bMET trials were male (57.64%), with an average age across trials of 24.92 (25.32 for MET and 24.83 for bMET)	Trials testing the effectiveness of MET planned 3–16 sessions (*M* = 7.60, median = 4). Only half of trials reported the dose delivered. Of those studies where dose was reported, an average of 90.96% of sessions were attended bMET utilised 1 or 2 sessions, with most treatment arms (57.89%) only planning one session. Across trials, 92.77% of participants completed the allocated dose of bMET (*M* = 1.24 and median = 1 sessions) Session duration for MET ranged from 60–90 min (*M* = 70, median = 60 min). Duration of bMET sessions was more varied, ranging 8.7–120 min (*M* = 51.76, median = 55 min)	All MET and 82.35% of bMET trials included cannabis use or diagnosis relating to cannabis use as an inclusion criterion In studies that provided data, the majority of randomised participants (87.79%) met criteria for a cannabis use disorder diagnosis On average, trial participants reported using cannabis on 19.32 (range 3.06–25.55) of the past 28 days Fewer than one third of trials reported the age of first cannabis use of their sample. The average age in those reporting was 14.42 (range 12.36–15.93)	***Strong Evidence****:* MET is effective in reducing frequency of cannabis use and dependence severity, although abstinence outcomes are less consistent [14, 26, 30]. MET treatment benefits were not maintained six to nine months post intervention when extended follow-up was examined [14] ***Mixed evidence:*** There is good evidence that briefer forms of MET (bMET) are effective in reducing cannabis use in clinical trials [14], however there is weaker evidence for the translation of bMET to primary care and emergency department settings [34]
Combined CBT + MET **(CBT + MET)**, and a brief variant of CBT + MET (**b(CBT + MET)**)	Of the trials that utilised combined CBT + MET, 7 were conducted in the USA, 4 in each of Europe and Australia, and one in Brazil. One trial pooled data from sites in the USA, Australia and UK Three trials implemented b(CBT + MET), two in the USA and in Australia. None were conducted in Europe Of all trials that utilised a form of CBT + MET, 55.00% recruited only adults (k = 11 CBT + MET). 40.00% of trials recruited a mix of adolescents and adults (k = 6 CBT + MET; 2 b(CBT + MET)). A single (5%) b(CBT + MET) trial recruited only adolescents On average, participants across trials were male (70.50%) and 23.49 years of age (24.83 for CBT + MET, 16.13 for b(CBT + MET))	Number of planned sessions of CBT + MET ranged 4–24 (*M* = 9.26, median = 9 sessions). Dose administered was reported in 60% of trials. Participants completed, on average, 72.04% of sessions that were planned (*M* = 6.06, median = 5.20 sessions attended) Trials implementing b(CBT + MET) implemented 1 or 2 sessions (*M* = 1.75 and median = 2). Only half of trials reported the number of sessions completed, with participants only attending an average of 97.50% of planned sessions, with average dose of 1.45 sessions (median = 1.45) Session duration for CBT + MET ranged from 50 to 95 min with an average duration of 69.58 min (median = 60 min) across trials. Only two of the b(CBT + MET) trials reported intervention duration, both at 60 min	All CBT + MET trials included cannabis use or diagnosis of a cannabis disorder as an inclusion criterion, with 95.36% of randomised participants meeting criteria for a cannabis disorder Of the b(CBT + MET) trials, 50% similarly included a cannabis exposure criterion, with the other studies sampling substance use more broadly. 81.70% of participants across all b(CBT + MET) met criteria for a cannabis disorder On average, trial participants reported using cannabis on 18.48 (range 7.55–25.82) of the preceding 28 days Just over a third (36.36%) of trials reported the average age of first use (*M* = 15.00, range 12.40–16.44)	***Strong Evidence:*** Combining CBT + MET is effective in reducing frequency of cannabis use and dependence severity, although fewer studies have examined abstinence as a primary outcome [14, 26, 30]. Combining CBT and MET may be more effective than either treatment approach individually [14]. Where extended follow-up was included, CBT + MET treatment benefits were not maintained at six to nine months post intervention [14]
Contingency Management **(CM)**, including as adjunct to CBT **(CBT + CM),** MET **(MET + CM)**, and combined CBT + MET **(CBT + MET + CM)**	11 trials examined CM alone or as adjunct to CBT, MET or combined CBT + MET. Of these 9 were conducted in the USA and one each in the UK and Canada All trials included only adults (average age of 28.71 years) The majority of participants recruited were male (78.68%)	CM has predominantly been tested as adjunct to CBT, MET or combined CBT and MET (CBT + MET). The duration of CM in these trials is anchored to the duration of the paired therapy, and ranged 3–14 sessions or weeks. In trials that reported attendance of CM sessions, participants completed 65.57% of those sessions Four trials examined CM alone, and ranged 9–12 weeks in duration. In trials that reported average attendance, participants attended 57.78% of the CM sessions that were planned	All trials had an inclusion criterion of cannabis use or diagnosis of a CUD All but three trials consisted entirely of participants who met criteria for a CUD. Across trials 94.03% of participants met diagnostic criteria for a cannabis disorder. This is due to the high prevalence of cannabis disorder as an inclusion criterion in trials that utilised CM The majority of trials reported cannabis use frequency, with participants using cannabis on an average of 19.82 of the preceding 28 days Fewer trials reported age of first use (k = 4; *M* = 14.74)	***Strong Evidence:*** Adding CM to CBT, MET, or CBT + MET reliably reduces frequency of cannabis use and reduces dependence severity, but further studies are required to confirm if adjunctive CM is effective in increasing abstinence rates [14] ***Limited evidence:*** There are currently not enough good quality studies to assess CM as a stand-alone treatment [14]
Family Therapy **(FT)**	Of the six trials conducted using FT, four were conducted in the USA and two in Europe (The Netherlands; and a trial spanning Belgium, Germany and The Netherlands) Half of trials recruited only adolescent participants, and the remaining half recruited a mix of adolescents and adults. Typically recruitment was limited to 17 or 18 years of age, although one trial included participants up to 35 years. The average age of participants across trials was 17.18 years Just over half of participants were male (55.15%)	Just over half of trials (66.67%) reported the number of sessions planned, ranging 12–20 (*M* = 14.75, median = 13.5). An average of 74.50% of these sessions were attended (average dose = 10.90 sessions) Session duration ranges from 60–180 min across trials	Half of trials included cannabis use or diagnosis as a criterion, the remainder sampled substance use more generally. In trials that reported data, 90.66% of participants met criteria for a CUD All but one trial reported frequency of cannabis use, with participants using cannabis on 17.88 of the preceding 28 days No trials reported the average age of first cannabis use for their samples	***Mixed evidence:*** There is mixed evidence that FT may be effective in younger populations, primarily due to the comparability of theoretical heterogeneity across studies. More FT studies are required in adult populations
Mindfulness Meditation **(MM)**	No trials evaluated MM in isolation. One trial conducted in the USA evaluated MM in combination with MET. The trial recruited an exclusively female sample of adults with an average age of 23 years	Two sessions of MM were planned, with all participants attending the first session and 73% attending the second, equating to an average dose of 1.73 sessions Duration of sessions was not specified	Inclusion criteria required participants to have used cannabis in the past three months. Whether participants met criteria for a formal cannabis use disorder was not specified Participants reported using cannabis on 16.50 of the preceding 28 days. Age of first cannabis use was not reported	***Limited evidence:*** There is currently limited evidence examining MM in isolation to assess efficacy in treating CUDs
Social Support counselling **(SS)**	Two trials, both conducted in the USA on adult samples, evaluated the effectiveness of SS in a group context The average age of participants recruited was 32.21 years, and 76.09% were male	Both trials planned ten sessions of group SS, with 75.70% of sessions completed by the enrolled participants Duration of sessions in both trials was 120 min	Both trials required participants to have used cannabis within the preceding months to qualify for study inclusion. No data was reported in either trial on the number of participants meeting diagnostic criteria for a CUD The average age of first cannabis use was 16.14 across the two trials	***Limited evidence:*** There are currently not enough good quality studies on SS to assess efficacy in treating CUDs
Drug Education counselling **(DE)**	Ten trials examined DE, 6 conducted in the USA, 2 in Europe (1 UK and 1 Switzerland), and 1 each in Canada and Australia Of the ten trials, none restricted recruitment to adolescents, whereas half restricted their sample solely to adults. Of the remaining trials, three recruited adults and adolescents, one had no age restriction, and the final trial did not state whether age was a criterion. The average age across trials was 22.34 years 64.89% of participants across DE trials were male	The number of DE sessions planned within treatment arms ranged 1–16 (*M* = 4.10, median = 1), with most (70.00%) trials utilising a single session. Across treatments, 85.99% of planned sessions were completed Only half of trials reported their session duration, which ranged 20–120 min	Nine trials evaluating DE specified cannabis use or a CUD as an inclusion criterion. A single trial specified general substance use as the inclusion criterion The majority of trials (60.00%) did not report the proportion of participants that met criteria for CUD. In those that did, the majority of participants (88.13%) met criteria for a CUD Only 30.00% of trials reported the age of first cannabis use, with an average across trials of 15.38 years	***Limited evidence:*** There are currently not enough good quality studies on DE to assess efficacy in treating CUDs
Relapse Prevention **(RP)**	Two trials conducted in the USA evaluated the effectiveness of group RP The trials recruited predominantly male (76.09%) adults with an average age of 32.21	Both trials planned ten sessions of 120 min each. Across trials, participants attended an average of 75.70% of the planned sessions	Both trials required participants to have used cannabis in the past three months, with participants reporting using cannabis on an average of 25.09 of the preceding 28 days. Participants’ reported age of first cannabis use was 16.14 years of age No data reported on the proportion of participants that met criteria for a cannabis use disorder	***Limited evidence:*** There are currently not enough good quality studies on RP to assess efficacy in treating CUDs
Mutual Help Groups **(MHG)**, based on the 12-step approaches	A single trial conducted in the USA evaluated the effectiveness of MHG. The trial recruited adults and adolescents (range 12–18) with an average age of 15.60. Participants were predominantly male (83.87%)	The number of sessions planned was variable, averaging 10–12, with an average dose of 6.8 sessions delivered. Session duration was not specified	Recent cannabis use and a primary cannabis use diagnosis were inclusion criteria for the trial. Frequency of cannabis use was not reported. Average age of cannabis initiation was 12.90 years	***Limited evidence:*** There are currently not enough good quality studies on Mutual Help Groups to assess efficacy in treating CUDs [14]

## Method

In this data synthesis we sought to extract pertinent study characteristics from 68 individual studies identified from recently published reviews. We provide a synthesis of study characteristics stratified along therapeutic approach by drawing on individual level study data, reported in Table 1. We focus on randomised controlled trials (RCTs) as they are the ‘gold standard’ in assessing efficacy and cause-effect relationships in addiction research [22]. RCT designs vary but what is consistent is that they have a control condition that is intended to exclude the possibility that the effect or association was caused by a third factor associated with both intervention and outcome. High quality RCTs apply blinding and random sequence generation to treatment and non-treatment/control groups; all groups have identical treatment exposure, except for the experimental group; and effect size is generated between the experimental and control groups to disentangle the specific power or efficacy of the focal experimental intervention [23].

We included RCTs that have been identified by peer reviewed and published systematic reviews and meta-analyses to ensure minimum quality of design, data, and findings. Despite drawing on largely the same body of work, these systematic reviews have reached inconsistent conclusions on the efficacy of these treatments. These inconsistencies are predominantly a consequence of incompatible categorisation of methodologies, interventions and participants characteristics across systematic reviews. By applying more detailed classifications, we can overcome some of the limitations of existing reviews. We provide a synthesis of study characteristics drawing on individual level study data, which provides new and novel insights into study characteristics stratified by treatment type. We relied on systematic reviews and meta-analyses as these are more rigorous than non-systematic narrative reviews in that they involve pre-determined criteria and quality requirements and a systematic extraction of the literature, avoiding the introduction of potential bias by including poor studies or studies favoured by the researchers [24]. The most recent systematic review on behavioural and pharmacological treatments for CUD was published in 2019 [25], with a review of systematic reviews published in 2021 [16]. In addition to these works we considered the systematic reviews published by Cooper et al. [26], Davis et al. [27], Gates et al. [14], and Halladay et al. [28].

## Results

### Effectiveness of behavioural therapies

A meta-analysis (10 RCTs) that pooled CBT, MET, CM and RP approaches showed an overall medium effect size (Hedges’ *g* = 0.44) in reducing cannabis use up to 14 weeks post treatment, compared to pooled control arms that consisted of inactive (i.e., waitlist) controls or active controls which contained no behavioural component (i.e., treatment as usual or psychological placebo) [27].

There have been eight reviews on behavioural interventions that aim to reduce problem cannabis use in individuals with and without CUD. These studies include adult and combinations of adult and adolescent populations. Three meta-analyses [14, 27, 28], three narrative systematic reviews [26, 29, 30] and one review of reviews [16] have analysed research on the effectiveness of separate psychosocial treatments in reducing cannabis use and promoting abstinence in adolescent and adults. There is also one meta-analysis [31] of psychosocial treatments for substance use more broadly in adolescents. Characteristics of 68 studies included in these eight reviews are summarised in Table 1.

These studies included stand-alone treatments defined by recognised theoretical principles and mechanisms (eg. CBT, MET including bMET, MM), adjunctive approaches that may add benefit to other psychosocial treatments (eg. CM) and selective components of more comprehensive psychosocial treatment approaches (eg. RP, SS). This review also defines psychoeducation and supportive counselling as psychosocial therapy (eg. DE, MHGs, based on the 12-step approaches such as MA) that may be incorporated into treatment with or without distinct theoretical principles (eg. CBT, MET). The aforementioned therapies were selected for this data synthesis because they were consistently reported across the existing reviews and are all recognised psychosocial therapeutic approaches. A limitation of this approach, however, is that it may fail to capture all treatment approaches that have been used to reduce cannabis use and CUD symptoms.

Psychosocial approaches for adolescents include individual, group, and family interventions (FT). A narrative systematic review of adolescent substance use disorder treatment did not examine outcomes for cannabis use separately [32]. Systematic reviews that selected only studies with adolescent samples are reported separately under *Adolescent behavioural interventions.*

### Characteristics and effectiveness of behavioural RCTs

A wide range of psychosocial approaches for individuals with CUD were included in systematic reviews. Of the studies identified in published systematic reviews, 15 included participants from European countries. The most widely examined behavioural interventions for cannabis were CBT, MET, and combinations of these two interventions. In studies where diagnostic data were available, the vast majority (89.71%) of study participants met either DSM or ICD criteria for CUD or cannabis dependence. The average duration of CBT was 12 sessions (67% of planned sessions delivered), MET 7.60 (91% of planned sessions delivered) and for combined CBT and MET 9.26 (72% sessions delivered). A brief form of MET that delivered only 1 or 2 sessions (average of 1.24) was evaluated in more trials than any other form of therapy. Participants in almost all trials were outpatients. Behavioural interventions were delivered primarily by clinical psychologists or psychiatrists, but most trials did not specify the training of staff delivering treatment.

Based on good quality studies, CBT and/or MET improve treatment outcomes for individuals with CUDs. At six months follow-up, treatment outcomes were similar between CBT and MET. Treatment gains were not usually maintained nine months post treatment in those studies that reported longer follow-up. CBT and MET (or combined CBT + MET) treatments that extend beyond four sessions over more than one month, appear to be more effective than fewer sessions over a shorter duration. If feasible, combining CBT or MET (or combined CBT + MET) with adjunctive CM reliably reduced frequency of use and cannabis problem severity, but more studies are required to assess if the same gains are achieved with abstinence goals. There is not enough current evidence to support use of RP, SS, DE, or MHGs in the management of CUDs.

### Adolescent behavioural interventions

Cannabis use typically commences in adolescence. Given the plasticity of the developing brain in adolescents, there is elevated risk for temporary and permanent neuropsychiatric changes with heavy use [35, 36]. Recent regulatory changes in countries that allow legal access to cannabis and the use of methods preferred by young people (eg. Cannabis infused lollies, drinks and vaping cannabis oils) may be exposing young people to increased harm [35–37].

Two systematic reviews have examined substance use treatment outcomes for adolescent populations specifically in studies between 2007 and 2013 (19 studies, 5 cannabis specific, 1 European sample) and between 2014 and 2017 (11 studies, 4 cannabis specific, 3 recruiting an exclusively European sample) [32, 38]. The research that is available is on treatments that use behavioural approaches modified from those used in adult populations and designed to more effectively engage family and peers. These typically include family systems-based treatments and group CBT. These reviews found that in outpatient settings, the strongest and most consistent evidence was for family-systems based therapy, individual CBT and MET [32, 38]. Later reviews of the literature by Winters, et al. [16, 39] supported these findings, and noted that clinical trials show some support for CM in adolescent populations but require further research. There may be additional benefit in adolescent treatment approaches that integrated CM and family-systems based approaches [16].

### Digital behavioural interventions

Digital mental health interventions delivered by computer, phones and tablets, that became more widely used during the COVID-19 pandemic [40], have the advantage of offering greater geographic access to CUD treatment. Five systemic reviews identified individual studies of exclusively digital interventions [41–44]. Beneria et al.’s meta-analysis [41] of 17 studies of adolescents and young adults (n = 3,525, mean age range 16.3 to 29.8, 52.4% male) included three studies from Europe. It found that online interventions for this age group did not significantly reduce cannabis use among people with CUD [41]. The authors noted that there was considerable heterogeneity among studies and that more recent studies that used structured interventions that specifically targeted CU had more positive effects. These observations are consistent with the review by Walukevich-Dienst and colleagues [44] that found women, but not men, benefited from online, personalised feedback programs for cannabis-related problems.

An earlier, non-age restricted meta-analysis by Hoch and colleagues [43] (n = 1,928) identified four high-quality studies (two in Europe, two in adolescents and two in general populations) that examined digital interventions for problematic cannabis users in non-clinical settings (mean age range 20.0 [combined arms] to 31.9 intervention/30.2 control, pooled gender not reported). Pooled analyses indicated that self-reported cannabis use was reduced significantly post digital intervention. The strongest treatment effects were reported in studies that used a web-based online chat with a trained psychotherapist. A subsequent non-age restricted meta-analysis (n = 2,963, average age range and pooled gender percentage not reported) including nine studies (one European) also found that computerized interventions were effective, for both self-reported use (eight studies) and biological verification via urine testing (one study) [45]. A larger meta-analysis with a broader age range (17–70) by Boumparis and colleagues [42] of 20 treatment digital interventions for cannabis users (n = 5,197) found that cannabis use was significantly reduced post-treatment (g = 0.12), but these treatment gains were not maintained at 12-month follow up.

A challenge of digital online interventions is to accurately identify from the original studies the type of behavioural treatment that is being used (see “Behavioural Therapies”). This may be largely due to the difficulties in fidelity testing across multiple, evolving electronic platforms and user interactions. More good quality studies are required, but the preliminary conclusions from existing quality studies are that the strongest evidence of efficacy in reducing problem cannabis use and CUDs is for computerized interventions that included personalised online feedback, offered computer-delivered MET or CBT, and were clinician-assisted.

### Pharmacotherapy for problem cannabis use, cannabis use disorder and cannabis withdrawal

Various classes of drugs have been trialled to treat problem cannabis use and/or withdrawal and associated symptoms [46]. These have included Δ^9^-tetrahydrocannabinol (THC) preparations (ie. cannabinoid agonists, eg. Nabilone, Dronabinol, Nabiximols, fatty acid amide hydrolase inhibitor PF-04457845), cannabinoid antagonists (eg. Rimonabant), cannabidiol (CBD) preparations, opioid antagonists (eg. Naltrexone), anticonvulsants (eg. Topiramate, Gabapentin, Quetiapine), glutamatergic modulators (eg. N-acetylcysteine), neuropeptides/hormones (eg. oxytoctin), nicotinic partial agonists (eg. Varenicline), antidepressants (eg. Escitalopram, Bupropion), mood stabilisers (eg. Lithium, Divalproex), non-benzodiazepine GABA(A) receptor agonists (eg. Zolpidem), Α2A adrenergic receptor agonists (eg. Guanfacine), antiemetics/antinauseants (eg. Aprepitant), anxiolytics (eg. Buspirone), cognitive enhancement agents (eg. Atomoxetine) and antipsychotics (eg. Clozapine, Ziprasidone).

### Evidence for pharmacotherapy

Findings from an earlier systematic review [47] have been supported by Cochrane meta-analysis [48], a combined narrative and meta-analysis systematic review of 26 RCTs [49] and a series of narrative reviews of studies [15, 30, 50, 51]. All conclude that that there is limited evidence that any pharmacological approaches effectively reduce problem cannabis use, treat CUD and/or withdrawal. A 2022 review [46] of medications used to treat cannabis withdrawal found that research in pharmacotherapy for cannabis withdrawal was limited by small patient numbers and low quality of studies. For example, of the 19 placebo-controlled studies reviewed, only three had more than 50 patients in the medication arm.

### Early Promising Findings

Pharmacotherapy for CUDs and withdrawal are less well developed than other drug use disorders but there are some promising results from small studies and/or studies that require replication. Replications may validate the practice of clinicians who use selected medications ‘off-label’ to treat cannabis use and/or withdrawal. Based on the available literature, the most widely studied and arguably most promising drug classes for problem cannabis use, CUD and cannabis withdrawal are cannabinoid agonist (ie. THC) preparations. Cannabinoid agonists are hypothesized to minimise cannabis withdrawal symptoms and reduce the patient’s motivation to use cannabis by occupying CB1 receptors. For example, male inpatients (46 active, 24 placebo) treated with the FAAH inhibitor (PF-04457845) and followed up as outpatients reported significant reduction in cannabis withdrawal in the first days of treatment and less cannabis use (self-report and urine THC-COOH concentrations) at four weeks follow-up [52]. On the basis of these initial positive outcomes, a large-scale multicentre study with a more diverse population using the FAAH inhibitor PF-04457845 is now underway (ClinicalTrials.gov Identifier: NCT03386487). The cannabis agonist Nabiximols (an equal ratio of THC and CBD) when combined with psychosocial treatment has shown reductions in cannabis use in cannabis dependent patients (n = 61 active, 67 placebo) up to 3 months post intervention [53].

CB1 antagonists (such as Rimonabant) have been shown in human experimental studies to block the effects of THC [54]. However, adverse clinical effects observed in Rimonabant trials included depression and suicidality that potentially reduced their clinical application and subsequent studies of this agent [55]. CB1 inverse agonists are being developed with fewer adverse effects, but their use has largely been restricted to preclinical studies. Other studies identified in the literature as showing early positive signs, despite weakness in the number or quality of studies, include opiate antagonists such as naltrexone (given the strong reinforcement mechanisms between opioid and cannabinoid systems), topiramate, N-acetylcysteine, gabapentin, oxytocin and varenicline. As of 2022, there were twelve (two in Europe) active studies investigating pharmacological treatments for CUD listed in the National Library of Medicine Clinical Trials Database.

In summary, no medications are currently approved to reduce adult cannabis use, CUD or cannabis withdrawal. Considerably fewer studies have been conducted in adolescent populations [56, 57] and a minority of existing pharmacotherapy studies have been conducted in Europe. Despite the current evidence, some medications, particularly cannabis agonists, are used ‘off-label’ in some international jurisdictions by a small number of prescribers. As with all prescribing, a comprehensive medical, medicine and drug and alcohol use history should guide the use and dose of these medications. All medications have side-effects and these need to be balanced against potential benefits from their unknown efficacy and largely untested safety in this population.

## Conclusions

Based on high quality behavioural studies, CBT and/or MET improve short-term treatment outcomes for individuals with CUDs but these gains are not usually maintained greater than nine months post treatment. If feasible, combining CBT or MET (or combined CBT + MET) with adjunctive CM reliably improves treatment outcomes. Typically, CBT and MET (or combined CBT + MET) treatments that extend beyond four sessions were more effective than fewer sessions over a shorter period. A small number of behavioural studies on cannabis intervention have been conducted in Europe. No medications are currently approved for use in adult or adolescent problem cannabis use, CUD or cannabis withdrawal. Few pharmacotherapy studies have been conducted in Europe. Despite the lack of current evidence, some prescribers use medications such as cannabis agonists ‘off-label’. Benefits of off-label prescribing need to be balanced against potential risk from their unknown efficacy and safety in cannabis using populations.
